# Dysregulated Expression and Subcellular Localization of Base Excision Repair (BER) Pathway Enzymes in Gallbladder Cancer

**DOI:** 10.22088/IJMCM.BUMS.7.2.119

**Published:** 2018-08-18

**Authors:** Manoj Kumar, Vijay Kumar Shukla, Pravas Kumar Misra, Mercy Jacob Raman

**Affiliations:** 1 *Cytogenetics laboratory, Department of Zoology, Banaras Hindu University, Varanasi, India.*; 2 *School of Biological and Environmental Sciences, Faculty of Basic Sciences, Shoolini University, Solan, Himachal Pradesh, India.*; 3 *Department of General Surgery, Institute of Medical Science, Banaras Hindu University, Varanasi, India.*; 4 *Departments of Pathology and Surgery, Indian Railways Cancer Institute and Research Centre, Varanasi, Uttar Pradesh, India.*

**Keywords:** AP endonuclease 1, DNA polymerase β, chronic cholecystitis, gallbladder cancer

## Abstract

Base excision repair (BER) pathway is one of the repair systems that has an impact on radiotherapy and chemotherapy for cancer patients. The molecular pathogenesis of gallbladder cancer is not known extensively. In the present study we investigated whether the expression of AP endonuclease 1 (APE1) and DNA polymerase β (DNA pol β), key enzymes of BER pathway has any clinical significance with gallbladder carcinogenesis. 41 gallbladder cancer, 27 chronic cholecystitis, and 3 normal gallbladder specimens were analyzed for the expression of APE1 and DNA polymerase β by western blotting, and subcellular localization studied by immunohistochemistry. The enzymatic activity of APE1 was also studied. The correlations with expression of the above proteins with clinical-pathological characteristics of gallbladder cancer patients were analyzed. The integrated density value ratio (relative expression) of total APE1 (37 kDa + 35 kDa variant) analyzed in the three groups of tissues, was 0.76±0.03 in normal gallbladder, 0.91±0.08 in chronic cholecystitis, and 1.12±0.05 in gallbladder cancer. APE1 was found to be up-regulated in 80% of gallbladder carcinoma samples (P = 0.01). A positive trend of APE1 expression with tumor stage and lymph node positivity was observed. The enzymatic activity of APE1 was found higher in gallbladder cancer samples in comparison with chronic cholecystitis. The integrated density value ratio of DNA polymerase β for normal gallbladder, chronic cholecystitis and gallbladder cancer tissue samples were 0.46±0.03, 0.7±0.06 and 1.33±0.1, respectively. DNA polymerase β was found to be upregulated in almost all gallbladder carcinoma samples (P =0.0001), and its expression was negatively correlated with age (P=0.02). DNA polymerase β expression showed a positive trend with tumor stage and nuclear differentiation of gallbladder cancer. It may be concluded that alteration of these BER pathway proteins may be the causal factors for carcinogenesis of gallbladder, and has targeted therapeutic potential.

Gallbladder cancer is a rare solid tumor with poor prognosis, and almost a lethal gastrointestinal malignancy with high prevalence in certain world populations mainly India, Chile, China, and Japan (1, 2). It has a median survival rate of 6 months, and 10% overall 5-years survival (3). About 70% of patients have a history of gallstone. Although there are many other known risk factors, gallstone is the most associated risk factor for this cancer (4). Since there is a lack of noticeable sign or symptoms, mostly patients are diagnosed at an advanced stage, and therefore it becomes incurable. Thus, no chemo or radiotherapy treatment has been established successfully for its advanced stage. Detailed knowledge of the molecular basis of this cancer is therefore desirable for early diagnosis and better therapeutic strategies for advanced stage.

DNA repair system maintains genomic integrity by detecting and removing DNA damage. ase excision repair (BER) pathway is one such pathway for the removal and repair of individual base damaged by various endogenous processes such as oxidation, alkylation, and deamination (5). AP endonuclease 1 is a multifunctional protein which is not only responsible for repair of AP sites but also acts as a redox factor maintaining transcription factors like p53, FOS, JUN, hypoxia inducible factor 1 subunit alpha (HIF-1α), paired box 5-6 (PAX5-6), and nuclear factor kappa B (NFkB), in an active reduced state (6). Another crucial enzyme of this pathway is DNA polymerase β which plays a role in single nucleotide gap filling in BER pathway downstream to AP endonuclease 1. DNA polymerase β can be distinguished from other DNA polymerases because of lack of associated poof reading activity (7). Probably because of error-prone features, DNA polymerase β accumulates mutations in the genome, and lead to genomic instability and cancer upon overexpression in the cell.

AP endonuclease 1 was reported to be elevated in gastric (8), ovarian, gastro-oesophageal, pancreatico- biliary cancers (9), medulloblastoma (10), and hepatocellular carcinoma (11). It has been shown that the knockdown of AP endonuclease 1 in cancer cell line reduces the cellular invasion and cellular doubling number, and makes the cancer cells sensitive to radiotherapy and chemotherapy by upregulating the oxidative DNA damage via NF-kB signaling pathways (12). DNA polymerase β was also found up-regulated in gastric (13), ovarian (14), and prostate cancers (15). It was reported that silencing this gene by siRNA, makes the cancer cell sensitive to chemotherapy (16).

There is limited information in gallbladder cancer regarding DNA repair pathway status and therefore, the present study has been undertaken with an aim to investigate the expression level of AP endonuclease 1 and DNA polymerase β in gallbladder cancer and to investigate the association with clinicopathological characteristics of gall-bladder cancer patients.

## Materials and methods

**Subjects and tissue samples**

All 71 tissue samples were collected from Sir Sunderlal Hospital, Institute of Medical Sciences-Banaras Hindu University (IMS-BHU), and Indian Railways Cancer Institute & Research Center, Varanasi, India between 2007 and 2011. The samples were immediately snap frozen after procuring from surgical unit of respective hospitals, and stored at -80°C for further work. The study groups consisted of 41 gallbladder cancer, 27 chronic cholecystitis, and 3 normal gallbladder tissue samples (Table 1). Normal gallbladder tissues were procured from three postmortem cases from the hospital as a control group for the study. Written consent was taken from all the patients and the study was approved by institutional ethics committee of IMS-BHU, and Indian Railways Cancer Institute and Research Centre, Varanasi. Histopathological reports of the patients were procured from the respective department of pathology of the hospitals.

**Protein lysate preparation**

Tissue lysate were prepared from frozen tissues of normal gallbladder**,** chronic cholecystits**,** and gallbladder carcinoma in radio immuno-precipitation (RIPA) buffer containing 50 mM Tris.Cl pH 7.4, 100 mM NaCl, 1 mM EDTA, 2 mM EGTA, 1 mM DTT, 1 mM PMSF, 1% Nonidet NP40, 0.1% SDS, and protease inhibitors cocktail (Roche, USA) by conventional method in mortar and pestle. Then the homogenates were kept on ice for 20 min for proper cell lysis. After centrifugation at 12000 rpm at 4°C for 15 min, supernatants were collected as a tissue protein lysate, and kept at -80 °C for protein estimation and western blotting. Protein concentration was estimated by Bradford’s assay (17).

**Western blotting**

Protein lysates were resolved by electrophoresis in 12% polyacrylamide gel and transferred on polyvinylidene difluoride membrane (Millipore) for overnight at 4 ºC. Blocking was done with 1% BSA made in Tris buffer saline containing 0.1% Tween 20 (TBST) for 2 h at room temperature. Then membrane was probed with anti-human DNA polymerase β (1:200) and anti-human AP endonuclease1 (1:2000), (anti-human DNA polymerase β and anti-human APE1, rabbit polyclonal antibodies are gift from Prof. Rajendra Prasad, NIEHS, USA) for overnight at 4 °C, and then blots were washed with TBST, and incubated with secondary antibody tagged with alkaline phosphatase. Nitro-blue tetrazolium and 5-bromo-4-chloro-3'- indolyphosphate (NBT/BCIP) (Sigma Aldrich, USA) were used as substrates to develop the signals. The same blot was re-probed with anti-actin mouse monoclonal primary antibody (1:3000) (Sigma Aldrich, USA) for equal loading, and then developed by using the above described method.

**Preparation of cell-free extracts for APE1 oligonucleotide incision assay**

Cell free extracts were made from chronic cholecystitis and gallbladder cancer tissue samples.

**Table 1:** Patients demographic and pathological features for gallbladder cancer

**Characteristics** **Age** (median age 53 years)	**Numbers**	**Percentage (%)**
≤53	16	39%
≥53	25	60.9%
**Sex**		
Male	10	24.3%
Female	31	75.6%
**Nuclear Differentiation**		
Well	24	58.5%
Moderate	12	29.2%
Poorly	5	12.1%
**Tumor stage**		
I	4	5.5%
II	13	33.3%
III	14	36.1%
IV	10	25%
**Metastasis**		
Yes (M1)	7	16.6%
No (M0)	34	83.3%
**Lymph node status**		
Yes (N1&2)	18	41%
No (N0)	23	58.3

Tissue samples were homogenized in buffer I containing 10 mM Tris-Cl, pH 7.4, 200 mM KCl, and protease inhibitors (Roche, USA/ 1 tablet for 10 ml). Equal volume of buffer II containing 10 mM Tris-Cl, pH 7.4, 200 mM KCl, 2 mM EDTA, 2 mM DTT, 0.2% NP40, 40% glycerol, and protease inhibitors were added and kept at 4 °C with shaking for 1 h. After centrifugation at 14000 rpm at 4 ^o^C for 10 min, supernatants were collected and frozen overnight. Next day, the homogenates were dialyzed in a buffer containing 50 mM HEPES pH 8.0, 0.1 mM EDTA at 4 ^o^C for 1h, and fresh buffer was added and kept for dialysis for overnight at 4 ^o^C. Aliquots were made and snap frozen in liquid nitrogen and stored at -80 ^o^C.

**Oligonucleotide incision assay (APE1 activity assay)**

The oligonucleotide incision assay was performed to estimate the APE1 incision activity by taking 45 bp DNA substrate containing “U” at its 21^th^ position (gift from Prof. Phyllis Strauss, Northwestern University, USA). 10 pmol of “U” containing oligonucleotide substrate was 5’-end labeled using T4 polynucleotide kinase (Fermantas Inc, USA) and 50 µCi of [γ-^32^P]-ATP, and the labeling reaction was incubated at 37 °C for 45 min in water bath, and after that the reaction was stopped at 70 °C for 10 min, and immediately the same concentration of complementary strand was added for annealing. Labeled oligonucleotide substrate was purified by sephrose G-50 column (Sigma, USA) and eluted labeled DNA substrate was kept at -80 °C for further use. Then time dependent experiments were performed i.e. 0 min, 3 min, and 6 min in duplicates by incubation of 400 nmol of labeled DNA substrate with 5 µg of cell free extracts of chronic colecystitis and gallbladder cancer tissues in reaction buffer in total volume of 50 µl. After that, reaction was stopped by adding 2 µl of 0.5 M EDTA pH 8.0. After purification by standard phenol: chloroform method, DNA was eluted and run in 12% urea polyacrylamide gel at 1500 V. After proper separation, the gel was dried in a gel dryer (Bangalore Genie, India), images were taken either on X-ray film or by phosphorimager (BioRad, USA).

**Immunohistochemistry**

The tissues were fixed in Bouin’s fixative, and then paraffin-embedded tissues were cut at 5 µm thickness. Paraffin sections were deparaffinized in xylene, and rehydrated through downgrade grades of alcohol, and washed in Tris buffer saline (TBS) twice for 5 min. Antigen retrieval was done in 10 mM EDTA pH 8.0 at 95 °C for 5 min in coupling jar, and then gradually cooled under running tap water. Endogenous peroxidase was blocked in 3% hydrogen peroxide made in 50% methanol for 5 min, and sections were washed in TBS. For 8OHdG staining, RNase and proteinase digestion was done for exposing epitope, and denatured in 0.1 N HCl for 5 min in coupling jar. Sections were washed with TBS twice for 5 min, and then blocked in blocking buffer for 2 h at room temperature, and incubated with primary antibody for overnight at 4 °C. Next day, sections were washed in TBST (containing 0.05%Tween 20, Sigma, USA) and incubated with working dilution of biotinylated secondary antibody for 2 h at room temperature. After washing with TBST, prior prepared avidin-biotin peroxidase complex (Vector laboratories IHC kit, USA) was applied for 45 min. Then sections were washed in TBST, and developed for 5 min using working 3, 3'-diaminobenzidine (DAB) solution as a substrate. The sections were counterstained in diluted heamatoxylin, and dehydrated in graded alcohol and xylene. Mounting was done in DPX with coverslip. As a negative control, primary antibody was substituted with blocking buffer.

**Statistical analysis**

For relative expression analysis, first the integrated density value (IDV) was calculated for each protein, and ratio was calculated by loading the control (level of actin) for respective samples. The expression level of AP endonuclease 1 and DNA polymerase β in normal gallbladder, chronic cholecystitis, and carcinoma gall bladder were compared by Mann-Whitney U test for statistical significance. Correlation coefficient (Pearson's correlation coefficient) test was used to examine the correlation between DNA polymerase β, and AP endonuclease 1 with age. The association between the expression level of APE1 and DNA polymerase β with clinical factors like tumor stage, nuclear differentiation, gallstone presence, lymph node involvement, and metastasis were analyzed by Mann-Whitney U test. A two-tailed p-value of less than 0.05 was considered as significant difference. All tests were carried out by SPSS 16.0 and Graph Pad statistical software.

## Results

**Expression profile of AP endonuclease 1**

**Fig. 1:**
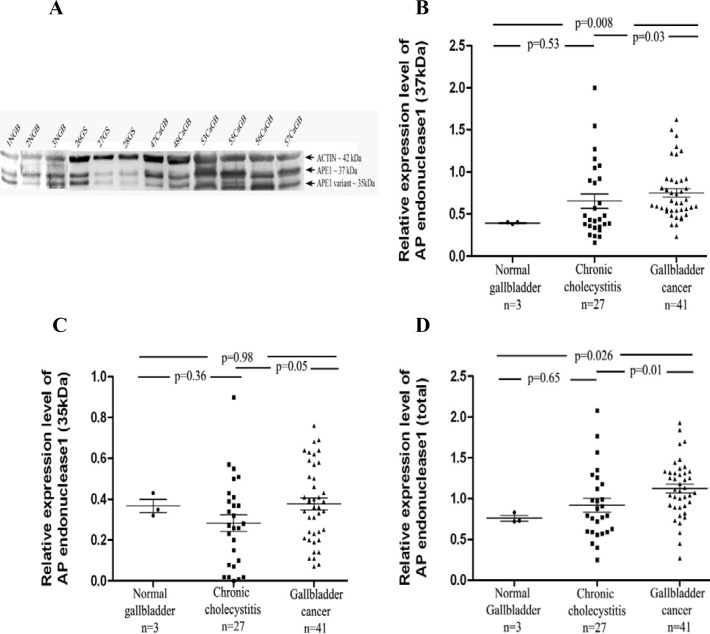
Protein level of APE1. A: APE1 protein levels in normal gallbladder, chronic cholecystitis and gallbladder carcinoma tissues assessed by western blotting; B: significant elevated APE1 (37 kDa) protein level in gallbladder cancer samples in comparison with normal gallbladder and chronic cholecystitis; C: no changes were observed in APE1 variant (35 kDa) protein level between gallbladder cancer and normal gallbladder tissues; D: the expression level of total APE1 protein in gallbladder cancer was significantly higer in comparison with normal gallbladder and chronic cholecystitis.

Two distinct bands of AP endonuclease 1 were observed in all types of gallbladder tissues examined at 37 kDa and 35 kDa position (Fig 1.A). The mean relative expression levels (IDV) of the regular form of APE1 (37 kDa) in normal gallbladder, chronic cholecystitis, and gallbladder cancer were 0.39 ± 0.01, 0.65 ± 0.45, and 0.75 ± 0.32 (mean ± SEM), respectively and for APE1 variant (35 kDa) were 0.36 ± 0.03, 0.28 ± 0.04 and 0.37 ± 0.02, respectively. Then we analyzed the total APE1 level in all the three groups of tissue samples, and the mean relative expression levels were 0.76 ± 0.03 in normal, 0.91 ± 0.08 in chronic cholecystitis, and 1.12 ± 0.05 in gallbladder cancer. In gallbladder cancer tissues, the amount of the long form of APE1 (37 kDa) was significantly higher when compared with normal gallbladder (P = 0.008) and chronic cholecystitis (P= 0.03) (Fig 1.B) while the relative expression level of the short form (35 kDa) was not significantly different in cancer tissues from the other two (Fig 1.C). The relative expression level of total AP endonuclease 1 (37 kDa + 35 kDa) was significantly higher in 80% of gallbladder cancer samples (32/41) when compared with normal gallbladder (P = 0.026) and also with chronic cholecystitis (P = 0.01) samples (Fig 1.D).

**Association of APE1 (total) expression with the clinicopathological characteristics of the gallbladder carcinoma**

**Fig. 2:**
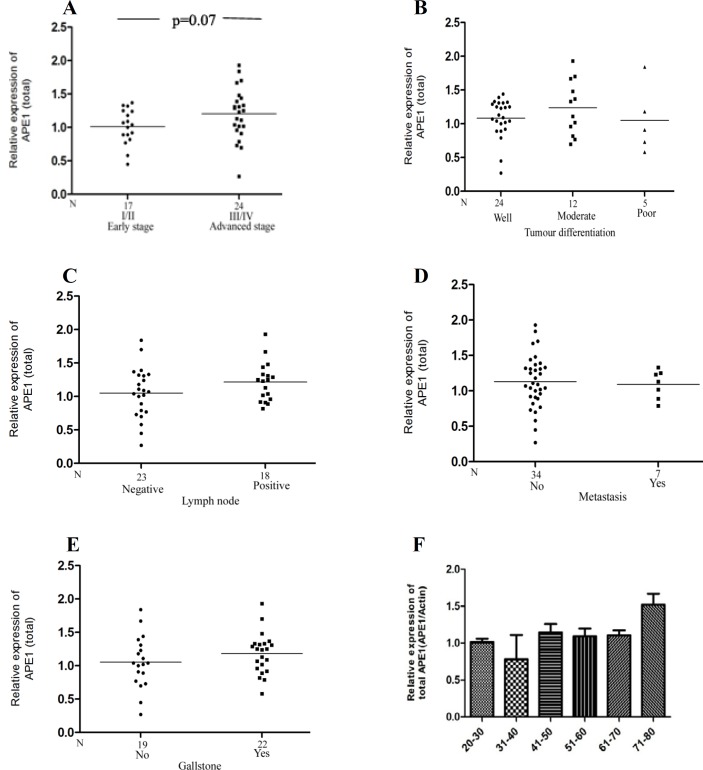
Association of APE1 protein level with clinicopathological factors in gallbladder cancer patients. A positive trend was noted between total APE1 protein levels with tumor stage (I/II vs III/IV) (A) and lymph node status (C), whereas no significant association was observed with (B) nuclear differentiation, (D) metastatic condition, (E) gallstone presence, and (F) age

A positive trend was noted in APE1 expression level with tumor stage (P = 0.07) (Fig 2.A) and lymph node involvement (P = 0.18), respectively, (Fig 2.C), though it was not significant. Higher expression was observed in higher tumor stage (III/IV), and also in moderately differentiated adenocarcinoma (Fig 2.B). The expression was higher in the gallbladder carcinoma patients having gallstone compared with gallbladder cancer patients without gallstone (Fig 2.E). There was no significant correlation between the expression level of total APE1 with age and metastatic condition of gallbladder cancer (Fig 2 D, F).

**Sub cellular localization of APE1**

**Fig. 3:**
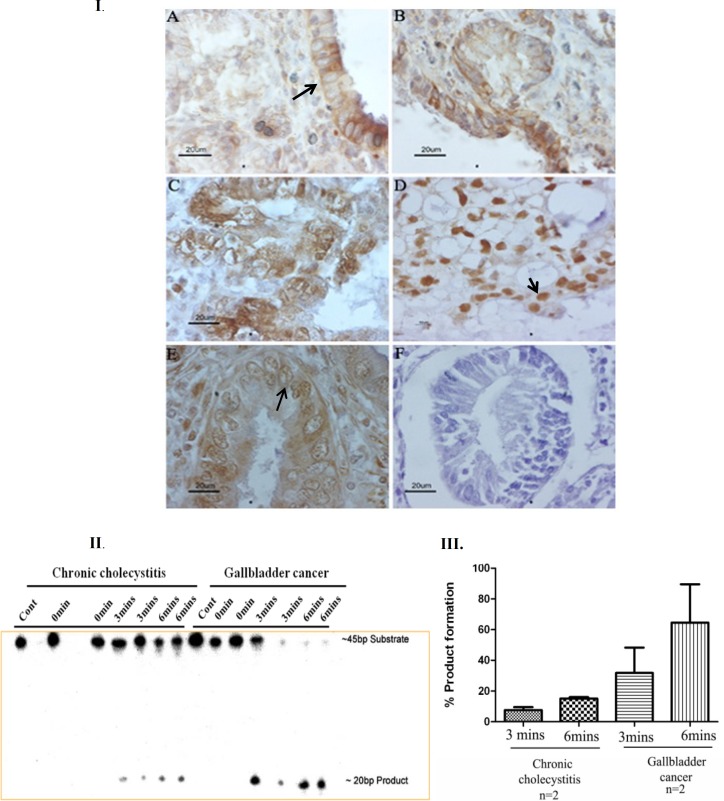
Immunohistochemical analysis of AP endonuclease 1 subcellular localization in paraffin sections. A: normal gallbladder; B: chronic cholecystitis; C, D & E: gallbladder adenocarcinoma; F: negative control for immunohistochemistry. Arrows indicate cytoplasmic (I.A), nuclear (I.D), and nucleocytoplasmic (I.E) localization. II: APE1 activity in chronic cholecystitis and gallbladder cancer samples. III: mean±SD of percentage of product formation in chronic cholecystitis and gallbladder cancer samples.

Immunohistochemistry of AP endonuclease 1 showed that all normal gallbladder (3) as well as chronic cholecystitis (10) showed cytoplasmic localization. No nuclear localization of APE1 was observed in normal gallbladder as well as chronic cholecystitis cases. While in case of gallbladder carcinoma, uniform nuclear staining was seen in 19 out of 40 cases (47.5%), uniform cytoplasmic staining was detected in 7 out of 40 cases (17.5%), and in the remaining 14 cases (35%) both nuclear and cytoplasmic staining was observed (Fig 3. I). Nucleo-cytoplasmic localization was observed in those cases which showed relatively higher protein level of APE1 in western blotting.

**Incision activity of APE1**

Elevation in APE1 protein level and mostly nuclear subcellular localization raised the question whether this dysregulation in localization alter the DNA repair activity of APE1. To answer this question, we checked the incision activity of APE1 in two chronic cholecystitis and gallbladder cancer samples, and we analyzed the percentage of product formation in duplicate. We observed high endonuclease activity in gallbladder cancer samples (n=2) compared with chronic cholecystitis (n=2) (Fig 3, II & III).

**Expression profile of DNA polymerase β**

**Fig. 4:**
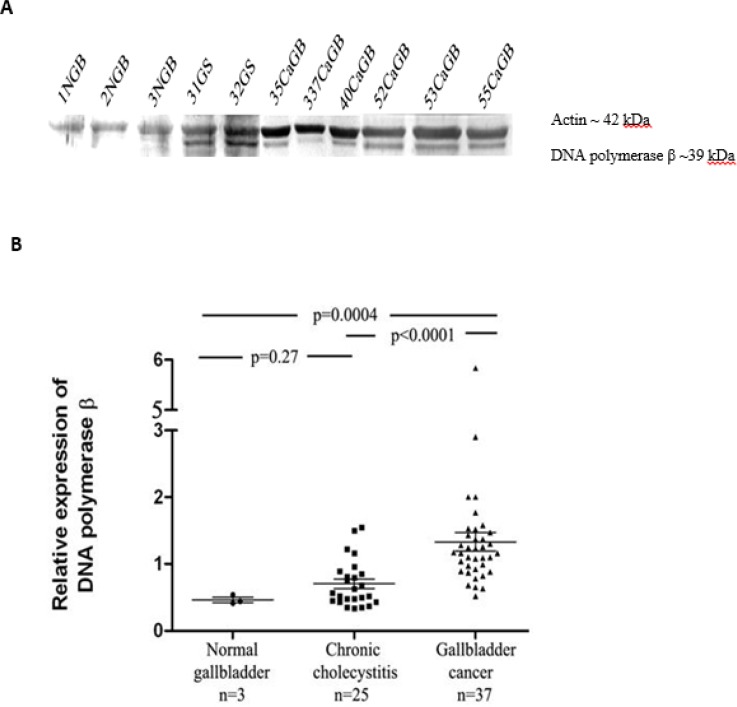
Expression levels of DNA polymerase β in gallbladder samples. A: protein levels of DNA polymerase β in normal gallbladder (NGB), chronic cholecystitis (GS), and gallbladder carcinoma (CaGB) tissues assessed by western blotting; B: the relative expression level of DNA polymerase β was significantly higher in gallbladder carcinoma samples compared with normal gallbladder and chronic cholecystitis

37 gallbladder carcinoma samples showed elevated expression of DNA polymerase β in comparison with chronic cholecystitis and normal gallbladder. We observed that the IDV ratio of DNA polymerase β were 0.46 ± 0.03, 0.7 ± 0.06, and 1.33 ± 0.1 in normal gallbladder, chronic cholecystitis and gallbladder cancer tissues, respectively. The relative expression level of DNA polymerase β was significantly up-regulated in gallbladder carcinoma tissue samples compared with normal gallbladder (P=0.0004) and chronic cholecystitis (P<0.0001) (Fig 4).

**Association of DNA polymerase β expression with the clinicopathologicals characteristics of the gallbladder carcinoma**

**Fig. 5:**
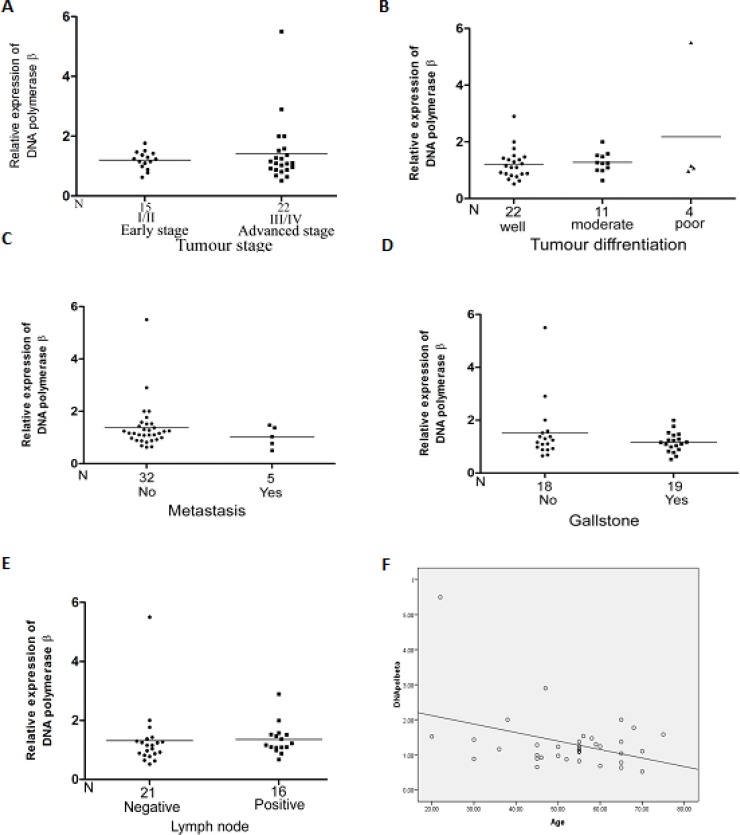
Association of DNA polymerase β protein level with clinicopathologicals factors of gallbladder cancer patients. There was no significant association between DNA polymerase β protein levels and (A) TNM stage (I/II vs III/IV), (B) Nuclear differentiation, (C) Metastatic condition, (D) Gallstone presence, (E) Lymph node status. Significantly negative correlation was found between DNA polymerase β expression with Age (F)

A significant negative correlation was found with the age of the gallbladder cancer patients and the expression of DNA polymerase β in cancer samples (P = 0.02). The expression level of DNA polymerase β was not found to be significantly correlated with the clinicopathologicals charac-teristic of gallbladder carcinoma (Fig 5).

**Sub cellular localization of DNA polymerase β**

**Fig. 6:**
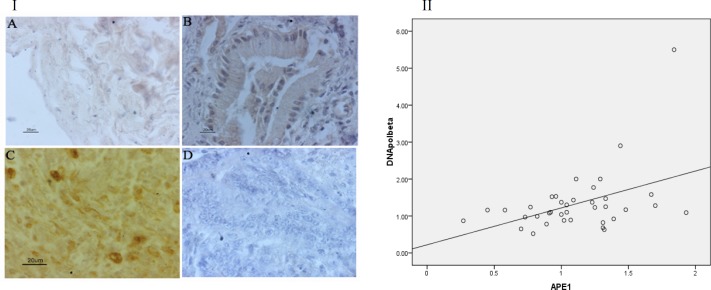
I: Immunohistochemical staining of DNA polymerase β. Cytoplasmic localization was observed in normal gallbladder (A) and chronic cholecystitis (B); nuclear staining was observed in gallbladder adenocarcinoma (C); D: negative control for immunohistochemistry. II: Correlation between the expression levels of DNA polymerase β and APE1 (total). Pearson correlation r = 0.36, P = 0.02

Cytoplasmic localization of DNA polymerase β was observed in the normal gallbladder (3) and chronic cholecystitis (5), but intense nuclear localization was observed in all gallbladder carcinoma tissues (5) (Fig 6.I). A significant correlation was observed between the expression level of APE1 and DNA polymeraseβ (fig 6.II) in gallbladder carcinoma(P=0.02) samples.

**Expression of 8-OHdG**

**Fig. 7:**
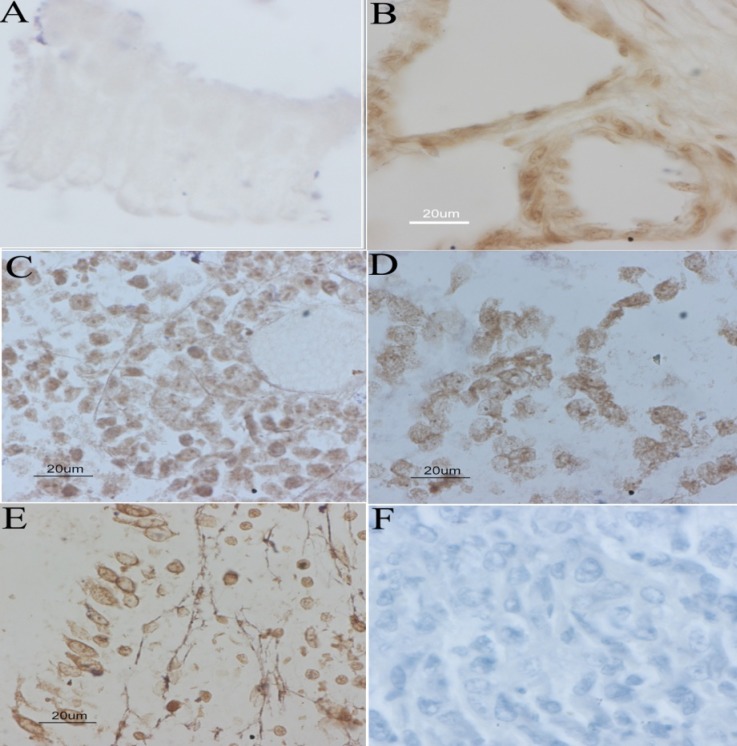
Immunohistochemistry of 8-OHdG in gallbladder tissue samples. A: faint staining in normal gallbladder; B: moderate staining in chronic cholecystitis; C, D and E: intense and homogeneous staining in gall bladder cancer; F: negative control for immunohistochemistry.

We observed that oxidative DNA damage was minimal in normal gallbladder tissue (3) and chronic cholecystitis (7), whereas in gallbladder cancer tissue samples (15), the intense nuclear staining of 8-OHdG staining was observed (Fig 7).

## Discussion

Accumulating evidence has demonstrated that over expression of BER proteins leads to tumor formation, less sensitivity towards radiotherapy and chemotherapy, metastasis, and a poor prognosis. However, clinical significance of APE1 and DNA polymerase β has not been previously analyzed in gallbladder carcinoma. In the current study, we demonstrated that APE1 was present in two forms in the gallbladder epithelial cell in normal as well as carcinoma samples. These forms of APE1 have different molecular weight i.e. 37 kDa and 35 kDa. Since single transcript was detected in human uterine smooth muscle tumor, so it was postulated that another form of APE1 (35 kDa) may be formed by post-transcriptional modification (18). In this study we observed that APE1 expression was elevated in 80% of the gallbladder cancer samples in comparison with chronic cholecystitis and normal gallbladder samples. Interestingly, we found that chronic cholecystitis showed higher expression level in comparison with normal gallbladder tissue. It may be concluded that the upregulation of APE1 in chronic cholecystitis may contribute to the carcinogenesis of gallbladder. In prostate carcinoma, the level of APE1 gets elevated from low in benign prostatic hypertrophy to high in prostatic intraepithelial neoplasia, and cancer (19). Several previous reports also demonstrated that the expression level of APE1 gets elevated in human solid tumors i.e. in pancreatic cancer (9), gastric cancer (8), germ cell tumor (20), cervical cancer (21), melanoma (22), and head and neck cancer (23). The expression of the long form of APE1 (37 kDa) was found to be elevated in gallbladder cancer samples. In human uterine smooth muscle tumor, the long form was elevated and its level was correlated with proliferating cell nuclear antigen levels, suggesting a correlation with increased proliferation (18). A positive trend was also observed in total APE1 expression level with tumor stage, with the highest expression in stage IV of the gallbladder cancer. It can be concluded that this higher expression may contribute to the selective growth of the tissues and suggests that APE1 may play a role in the progression of carcinogenesis of the gallbladder. In the present study, we could not find the correlation between APE1 expression level and tumor differentiation, but its expression was higher in moderately differentiated adenocarcinoma and in lymph node metastasis. Immunohistochemical studies of APE1 in gallbladder tissues revealed that APE1 was localized in cytoplasm in the epithelial cells of normal gallbladder and chronic cholecystitis but in gallbladder carcinoma, most of the cases showed nuclear and nucleo-cytoplasmic localization. Several studies on localization of APE1 in human cervical cancer like tissue (21), non-small cell lung cancer (24), bladder cancer (25) and squamous cell head and neck cancer (23), reported an intense up-regulation at the nuclear level. It has been reported that APE1 expression level and sub cellular distribution may be used as a predictive marker to indicate the sensitivity towards radio- or chemotherapy (26). Very interestingly, we observed that the enzymatic activity of APE1 was higher in gallbladder cancer in comparison with chronic cholecystitis, and correlated with protein level in tissue samples.

In the present study, almost all samples of the gallbladder cancer showed up-regulation in the expression of DNA polymerase β. Earlier studies revealed that DNA polymerase β level was high in cases of ovarian (14), esophageal (27), and colorectal cancer (28). DNA polymerase β has the lowest fidelity among other types of DNA polymerases of the cell. Overexpression of DNA polymerase β in human cancers leads to aneuploidy, mutations, abnormal localization of centrosomal associated gamma-tubulin during mitosis, increased microsatellite instability, and promotes tumorigenesis (14). We found a nuclear localization of DNA polymerase β in gallbladder cancer, whereas in normal gallbladder, a very faint expression was seen in the cytoplasm as observed in chronic cholecystitis. This suggests that high expression and nuclear localization of DNA polymerase β in gallbladder cancer may lead to functional deficiency of BER pathway, and induce carcinogenesis.

We have demonstrated that the level of DNA polymerase β decreases with the age of the patients affected with gallbladder cancer. This suggests that age and impairment in the DNA repair system has some links, and increase the susceptibility towards cancer. Earlier report suggests that p53- dependent response to DNA damage is altered with age, and also the level of certain critical DNA repair genes decline significantly with age (29). This alteration in the DNA repair genes predisposes the individual to mutagens, and leads to certain types of cancer.

In conclusion, we have shown that AP endonuclease 1 and DNA polymerase β get upregulated in the gallbladder cancer and also in chronic cholecystitis in comparison with normal gallbladder. Moreover, our study suggests that these BER proteins may be potential therapeutic targets in this cancer.
